# Medical History from the Earliest Times

**Published:** 1892-04-09

**Authors:** E. T. Withington


### Medical History from the Earliest Times.

By E. T. Withington, M.A., M.B. Oxon.

### III.—MEDICINE AS PRACTISED BY UNCIVILISED MAN.

It may be well to preface our survey of Medical History by a brief glance at the practice of the art by barbarous races, for we shall thus not only obtain some idea of the probable character of primitive medicine, but shall also find traces of many beliefs and practices which have survived under modified form in civilised communities. Only the briefest sketch of this vast subject can be here attempted.

In striking contrast to that tendency to materialism not altogether unjustly attributed to modern medicine, savage theories of disease are typically spiritualistic. This seems to be due partly to the habit, common to savages and children, of attributing life to everything, partly to the inherent nature of the subject. Disease and death, whatever they are, affect the inmost being of man, and even the most degraded savage holds that his inmost being is spirit, not body. How can merely physical agencies affect a spirit? Why should a simple blow on the head drive the soul from the body? The question has puzzled one of the greatest of physicans. "If Plato were alive," writes Galen, "I should like to ask him why great losses of blood, a draught of hemlock, or a severe fever, should separate soul and body, for according to Plato, death takes place when the soul removes herself from the body." If the savage does not perceive a difficulty, it is because he at once explains it by a supernatural theory of disease. Sometimes this theory is carried out to its fullest extent. Thus the Abipones hold that man is naturally immortal, and that even those killed in battle die not from their wounds, but from the enchantments of the hostile medicine men: if there were no medicine men, there would be no death. Even when the logic of facts has compelled the belief that clubs and arrows can kill without the aid of sorcery, faith in the spiritual origin of diseases remains unaltered. The supernatural agencies which cause them, are, however, of the most varied kind. Thus we may distinguish (i) independent disease demons; (ii) human enemies, who may act either by means of spirits of disease, over whom they have acquired authority, or by their own supernatural powers; (iii) the spirits of the dead; (iv) the spirits of animals killed in hunting. The belief in diseases caused by a good but angry divinity, who is to be appeased by prayer and sacrifice, belongs to a different and higher stage of civilisation. For examples of these various forms of belief the reader must refer to other sources, but some of the customs founded upon them, which are of medico-historical interest, may be here considered.

The savage medicine-man, who treats his patient on the excellent principle of removing the cause of the disease, has discovered at least three different methods of getting rid of a demon. First, the body of the patient may be rendered an unpleasant abode for the intruding spirit, and that in many ways. The sufferer may be vigorously squeezed and pommelled, beaten, starved, fumigated by evil-smelling substances, or be given nauseous medicines, which are especially useful if they act as emetics. To the first of these practices we may, perhaps, attribute the origin of that more systematic rubbing, undoubtedly of great antiquity, which has been re-introduced into scientific medicine under the name of "massage." Of fumigation we find a typical instance in the Apocrypha, where Tobit frees his bride from a demon by fumigating her with a fish's heart and liver, "the which smell, when the evil spirit had smelled, he fled into the uttermost parts of Egypt." Nor is this unconnected with our present subject, for we shall find that the mediæval physician looked upon Tobit's angel guide as his especial guardian, and that many of his drugs were well calculated to produce effects similar to those of the famous fish liver. It is not impossible that the belief, so common among hospital patients, that a "strong" medicine must, of necessity, be a very nauseous one, may be traceable to a similar origin.

But there is another and a less unpleasant mode of expelling a demon; he may be provided with a more appropriate dwelling place. Thus, in the Vedas, those most ancient monuments of Aryan literature, the demon of jaundice is entreated to depart into a yellow bird, and the chilly spirit of ague to take up his abode in the frog, while in later times the very site of the golden oriole was considered sufficient to cure the former disease. We may be able to trace a relation between this form of the demonic theory, and the mediæval docrine of "signatures," and shall perhaps find it not altogether unconnected with the still more famous medical dogma "Likes are cured by likes." Thirdly, there are persons endowed with power over the spirits of disease, who are able by charms and incantations to expel them from the patient's body, and in them we may not improbably see the first germ of the medical profession. It is usually asserted that the first physicians were priests; it would perhaps be equally true to say that the first priests were physicians. The savage appears to derive his ideas of higher existences, not so much from the starry heavens above, or the moral law within, as from those powers of nature which affect him physically, and especially from those which cause disease, and in his dealings with this spirit world, he looks primarily to those who, as he believes, can prevent or cure disease; his priests, in short, are "medicine men."

But may not those who have acquired such power over the spirits of sickness cause diseases as well as cure them? This brings us to that belief in witchcraft, the universality and terrible results of which amongst uncivilised races, is one of the saddest revelations of modern anthropology. We cannot here do more than mention this important aspect of the demonic theory of disease, nor need we trace it to its modern development. It is no curious old belief dug up by antiquaries to interest the student, or amuse the "general reader," but beyond parallel the most terrible delusion which has ever afflicted mankind. Within one century in Christian Europe alone, it brought torture and death to many thousands of innocent beings, and the amount of physical and mental agony caused by the belief in witchcraft through the long ages of man's early development passes all human estimation.

That diseases are often ascribed to departed spirits is a fact familiar to all students of folk-lore, and we shall meet with a curious instance of it in discussing the practice of medicine in ancient Egypt.

The supposed disease-producing power of the spirits of animals affords the simplest explanation of one of the most extraordinary customs of uncivilised man, that of the "couvade," or of the husband's lying-in when his wife has a baby. This strange habit, first noticed by Diodorus and Strabo as occurring in Corsica and the Pyrenees, has been found by modern observers among primitive races in every quarter of the globe. That a mother and her new-born child are peculiarly exposed to the attacks of evil spirits is a very wide-spread belief, originating, perhaps, in their great liability to diseases, and we find that in some races the husband refrains from hunting altogether at these times, lest the spirits of the injured animals should attack the helpless infant. It seems not impossible that he might sometimes go a step further, and, finding himself debarred from his usual occupation on these interesting occasions, conclude that he must in some way be seriously affected by the event, and proceed to treat himself accordingly.

